# Three Unrelated Patients of Roma Ethnicity from a Single Center Carrying the Same Deletion in *MYD88* Gene: A Founder Effect?

**DOI:** 10.3390/life15010020

**Published:** 2024-12-28

**Authors:** Roberta Romano, Francesca Cillo, Laura Grilli, Alessio Ciaccio, Lorenzo Bufalo, Elisabetta Toriello, Antonio De Rosa, Carmen Rosano, Emilia Cirillo, Giancarlo Blasio, Marika Comegna, Carmela Di Domenico, Giuseppe Castaldo, Claudio Pignata, Giuliana Giardino

**Affiliations:** 1 Department of Translational Medical Sciences, Pediatric Section, Federico II University of Naples, 80131 Naples, Italy; 2 Centre for Advanced Biotechnology (CEINGE), 80131 Naples, Italy

**Keywords:** inborn errors of immunity, founder effect, pyogenic infections, MyD88, next generation sequencing

## Abstract

MyD88 deficiency is a rare inborn error of immunity (IEI) characterized by susceptibility to pyogenic infections without overt signs of inflammation. Half of the reported patients belong to Roma descent, an itinerant ethnic group living mostly in Europe, with an increased risk of childhood mortality due to limited access to healthcare services. We describe three unrelated patients from the Campania region in Italy with MyD88 deficiency, all belonging to Roma descent and displaying severe or recurrent infections in early infancy. They underwent a comprehensive immunological work-up including targeted next-generation sequencing for IEIs that identified a homozygous pathogenic in-frame deletion c.157_159del p.(Glu53del) in *MYD88* gene, already described in this ethnic group, suggesting a founder effect. A high level of alert should be kept in patients of Roma ethnicity with early onset severe infections. Moreover, being associated with increased Immunoglobulin E (IgE) levels, this condition should be included in the differential diagnosis of Hyper-IgE syndromes.

## 1. Introduction

**Figure 1:**
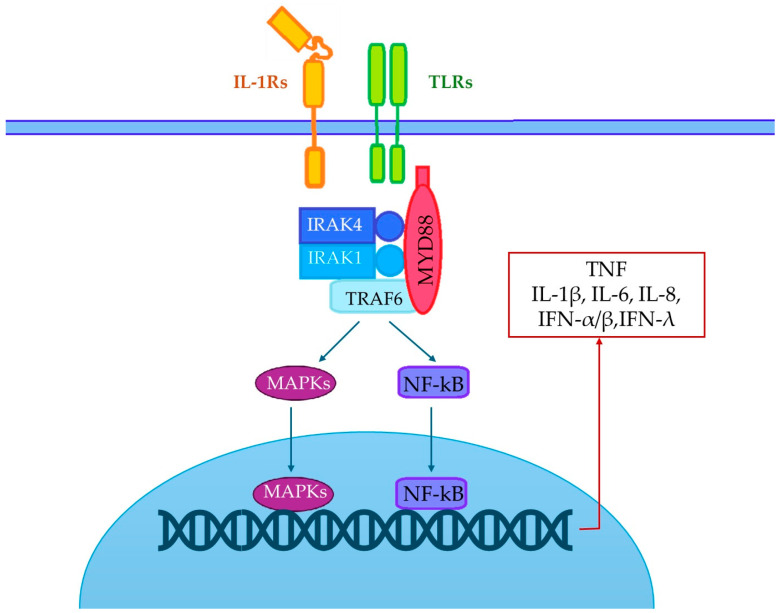
MYD88 and IRAK signaling pathway. Upon activation of transmembrane innate immunity receptors, IL-1Rs and TLRs (except on TLR3), the cytosolic adaptor MYD88 recruits the IRAK complex, and together, they activate mitogen-activated protein kinase (MAPK) and nuclear factor-kappa B (NF-κB) leading to the signaling and the expression of proinflammatory cytokines.

Myeloid differentiation primary response gene 88 (*MYD88*) encodes for a cytosolic adaptor downstream, a crucial pathway of innate immune response involving toll-like receptors (TLRs) and interleukin-1 receptors (IL-1Rs) [1]. It interacts with TLRs and IL-1Rs through a shared toll and IL-1R (TIR) domain and recruits interleukin-1 receptor-associated kinase (IRAK) complex, consisting of two non-catalytic subunits (IRAK-2 and IRAK-3/M) and two active kinases, IRAK1 and IRAK-4. The activation of the TIR-dependent pathway involving MyD88 and IRAK-4 leads to the synthesis of key inflammatory cytokines, such as TNF, IL-1β, IL-6, IL-8, IFN-α/β and IFN-λ (Figure 1) [2].

Therefore, MyD88 and IRAK-4 deficiencies underlie two rare autosomal recessive inborn errors of innate immunity sharing an indistinguishable clinical and immunological phenotype, thus representing phenocopies [3]. Their deficiency is characterized by a susceptibility to systemic and invasive pyogenic infections, mostly caused by *Streptococcus pneumoniae*, *Staphylococcus aureus*, *Pseudomonas aeruginosa* and *Shigella* spp. [4,5,6]. Typically, the highest mortality rate is observed in the first decade of life. Altered inflammatory response and absent increase in acute phase reactants may delay the identification of severe, potentially life-threatening infections that may present with few symptoms and no fever [4,7]. For this reason, these patients may die before receiving a diagnosis. In turn, this may affect the estimation of the incidence. Early identification and initiation of a proper treatment based on immunoglobulin replacement therapy (IgRT) and prophylactic antibiotics in the first decades of life may improve their survival [8].

Thereafter, a natural improvement in the disease course around the time of adolescence has been observed, posing doubt on the appropriateness of hematopoietic stem cell transplant (HSCT) despite the high mortality rate. As for MyD88 deficiency, nearly half of the reported patients belong to Roma descent, an itinerant ethnic group living mostly in Europe [9,10]. Due to their lifestyle, this ethnic group is at increased risk of childhood mortality deriving from limited access to healthcare services, substandard housing and recurrent migration, and these aspects may cause underestimation of the risk of underlying disorders in case of severe infections [11]. Conversely, genetic diseases of the immune system should always be considered since this group represents a genetic isolate. Herein, we report a case series of three unrelated patients from the Campania region in Italy carrying the same homozygous pathogenic in-frame deletion c.157_159del p.(Glu53del) in the *MYD88* gene.

## 2. Materials and Methods

The study IEI-Net 143, Project PNRR-MR1-2022-12376594, was approved by the Institutional Ethics Committee. Written informed consent was obtained from the patients or parents/legal guardians. The study was conducted according to the Code of Good Clinical Practice and to the ethical principles of the Declaration of Helsinki. Clinical and laboratory information was recorded through case report form. Detailed information concerning age, sex, age at onset of clinical manifestations and diagnosis, overall clinical status, and medical history were collected. For all three patients, laboratory information included complete blood count, total immunoglobulin count, Hepatitis B surface antibodies, anti-pneumococcal antibodies, total immunoglobulin E (IgE), lymphocyte subpopulations, Nitroblue tetrazolium chloride test (NBT), dihydrhodamine 123 test (DHR), mitogen proliferative response assay using phytohemagglutinin and pokeweed mitogens, toll-like receptor stimulation assay using poly I:C on peripheral mononuclear blood cells. For Patient 1 (P1), already described by Giardino et al. [10], genome sequencing has already been detailed in the previous manuscript. For Patient 2 (P2) and Patient 3 (P3), next-generation sequencing (NGS) analysis was carried out on exonic sequences of 5228 genes associated with known diseases enriched using SureSelect (Constitutional panels, Agilent Technologies, Santa Clara, CA, USA) for Illumina multiplexed sequencing. The bioinformatic analysis was restricted to a virtual targeted NGS (t-NGS) panel of 104 genes associated with the inborn errors of innate immunity and to a panel of 129 genes associated with Hyper-IgE, complement system and immune dysregulation disorders (list available upon request). A total of 50 ng of gDNA was processed for the analysis. For each gene, we analyzed the coding regions, 50 bp in each of the intronic boundaries, the promoter and the 3UTR for a total target size of about 1 Mb. Sequencing reactions were carried out on the MiSeq instrument (Illumina, San Diego, CA, USA) using a PE 150 2 flow cell, running 16 samples for each sequencing run to obtain an average coverage of about 200 (>95% of the gene’s target nucleotides are covered at >100 reads, with mapping quality score (MQ > 30) reads); 96% of the analyzable target regions were covered by at least 50×. The Alissa Align & Call version 5...2.10 tool (Agilent Technologies, Santa Clara, CA, USA), using the genome build hg19 as a reference, was used to perform alignments, variant calling and quality filtering. The median QV bases used in variant calling was 39, with an average read length of 141 bp. Variant filtering and interpretation were performed using Alissa Interpret version 5.2.6 CE IVD software (Agilent Technologies, Santa Clara, CA, USA), using GRCh38.p2 and annotation sources like 1000 Genomes (Phase 3 release v5, 10 September 2014, including GRCh38 data), ClinVar (NCBI ClinVar October 2019), DGV (Database of Genomic Variants, version 15 May 2016), ESP6500 (variants in the ESP6500SI-V2 dataset of the exome sequencing project, annotated with SeattleSeqAnnotation137), ExAC (ExAC release 1.0—including GRCh38 from lift-over data), OMIM (OMIM, version 25 October 2019), dbNSFP (dbNSFP v3.0b2: Database on functional predictions for nonsynonymous SNPs), dbSNP (dbSNP build 151), and gnomAD (gnomAD release 2.0.2). Sanger sequencing was used to confirm both the diagnosis and the carrier status of the parents.

A low-resolution family screening for human leukocyte antigen (HLA) typing was performed in P2 and P3, as well as their parents and siblings. DNA samples extracted from peripheral blood were analyzed using polymerase chain reaction (PCR) amplification and subsequent hybridization with sequence-specific oligonucleotide probes (PCR-SSO) and PCR amplification with sequence-specific primers (PCR SSP) at six loci: HLA-A, -B, -C, -DRB1, -DQB1/A1 and -DPB1.

## 3. Results

All the patients were of Roma descent. Among them, P1 and P3 were born from consanguineous parents. They all displayed infections in the first months of life. However, age at diagnosis was heterogeneous (Table 1). During the infectious episodes, all the patients showed only low-grade fever and persistently negative inflammatory markers.

Regarding P1, already described by Giardino et al. [12], her family history was positive for three siblings who had died at an early age, two due to meningitis and one of enteritis. At 7 months of age, she experienced an intestinal occlusion with stenosis of the ileocecal valve and mesenteric adenitis due to *Yersinia enterocolitica* infection, followed by recurrent episodes of granulomatous suppurative lymphadenitis with multiple localizations (axillary, cubital, thoracic and maxillary) due to *Staphylococcus aureus*, requiring surgical excision, starting at 8 months. Due to the poor knowledge of the condition, the diagnosis was obtained after almost 2 years of follow-up through the t-NGS panel for inborn errors of immunity (IEIs). Soon after the diagnosis, she was started on prophylactic antibiotic treatment with cotrimoxazole, with a dramatic improvement in the clinical course. She eventually died at the age of 7 years, almost one year after the discontinuation of the follow-up and the prophylaxis due to pneumococcal meningoencephalitis.

P2 had a longer medical history, beginning at age 2 months, with an endophthalmitis by *Pseudomonas aeruginosa* that caused ocular enucleation. Subsequently, several life-threatening infections ensued in his first 7 years of life, including pneumonia with pleural empyema, systemic Shigellosis, multiple and recurrent cutaneous abscesses positive for *Pseudomonas aeruginosa*, a retropharyngeal abscess, meningoencephalitis due to *Streptococcus pneumoniae* and multiple invasive abdominal abscesses. At the age of 6 years, due to the suspicion of an IEI, a first-line immunological work-up, including complete blood count, immunoglobulins, IgE and vaccine response, was performed. Due to the identification of increased IgE levels (1060 IU/L) and National Institutes of Health (NIH) Hyper-IgE Syndromes (HIES) score of 30, STAT3 haploinsufficiency was suspected, but it was ruled out through direct genetic analysis. At that time, he was in follow-up at another center, and no further immunological investigation was performed. Two years later, he was referred to our center where, based on his clinical history, ethnicity and previous experience with P1, MyD88 deficiency was immediately suspected and confirmed through t-NGS for IEIs. The patient was started on prophylactic antibiotic treatment and IgRT (Table 1) with an initial improvement in the incidence of infectious episodes that were managed with aggressive antibiotic treatment at the first signs of infection, even in the absence of overt clinical symptoms and laboratory markers of inflammation. Nevertheless, compliance with clinical monitoring and prophylaxis soon became insufficient, and so, considering the pre-existing severity of the case, we decided to perform HLA testing on the patient, siblings and parents that retrieved an identical donor in the mother. However, it should be noted that we only performed a low-resolution screening analysis that has a lower reliability compared to high resolution. Nonetheless, we did not perform a high-resolution analysis since the family refused this therapeutic option, and currently, the patient is lost on follow-up.

P3 was admitted to our institution at 2 years of age with a history of recurrent, suppurative sub-mandibular lymphadenitis, unresponsive to antibiotic treatment and requiring surgical drainage that had started at 2 months of life. His clinical history was also remarkable for recurrent otitis, and a liver abscess was identified at the abdominal ultrasound. Molecular testing revealed MyD88 deficiency and Glucose-6-phosphate dehydrogenase deficiency, the latter confirmed by enzyme assay. Due to this concomitant diagnosis, amoxicillin instead of trimethoprim–sulfamethoxazole was chosen as a prophylactic antibiotic, along with IgRT (Table 1), dramatically decreasing the incidence of infectious episodes. HLA low-resolution testing revealed an identical donor in the mother of the patient. Similarly, we did not carry out a high-resolution analysis on this patient since we ruled out HSCT due to the complete adherence to the prophylactic antibiotic regimen and Ig replacement therapy, as well as overall good clinical conditions. The immunological features are summarized in Table 2.

**Figure 2:**
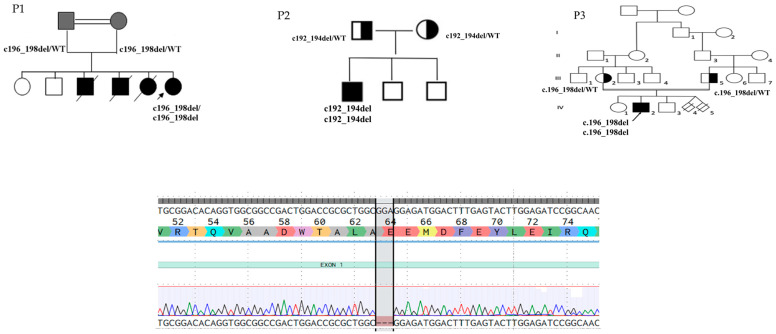
Patients’ family history and gene mutation. In the (**upper part**) of the figure, family pedigrees in which the probands are indicated with an arrow, the shaded shapes represent affected individuals, squares represent males, and circles represent females. Deceased siblings are indicated by line crossing. In the (**bottom part**) of the figure, genome sequencing analysis reveals the homozygous 3 nucleotide in-frame deletion NM_002468.5 c.157_159del p.(Glu53del) in exon 1 of *MYD88* gene.

The work-up of all our patients showed normal total white blood cell count (except for P1 who developed recurrent severe post-infectious neutropenia), serum IgA and IgG levels within normal ranges with slightly decreased IgM and increased total IgE (P1: 1190 IU/l; P2: 2014 IU/l P3: >2000 IU/l), and normal immunophenotyping (including adhesion molecules, CD11a/b/c/CD18). Hepatitis B surface antibodies were not detectable for P2 and P3, present in P1; anti-pneumococcal antibodies were absent in all three patients. Proliferative response to mitogens was normal. NBT and DHR 123 tests were performed to rule out Chronic Granulomatous Disease, based on the clinical phenotype of recurrent suppurative infections, turning out normal for all, whereas cytokine production after in vitro poly I:C TLR stimulation assay was altered in all three. t-NGS panel for IEIs identified a homozygous known pathogenic in-frame deletion c.157_159del p.(Glu53del) NM_002468.5 as of latest ClinVar annotation (formerly known as [c.192_194del GGA (p.Glu66del)], and [c.196_198del GAG (p.Glu66del)]) in *MYD88* gene in all the three patients, subsequently confirmed by direct Sanger sequencing. Already described in several affected families and in at least four Roma descent patients from the literature [10], this deletion removes a single conserved glutamic acid residue in the Death domain, resulting in greatly diminished protein levels (Figure 2).

## 4. Discussion

We described three patients of Roma ethnicity, belonging to unrelated families, with early onset pyogenic invasive infections, managed according to the latest evidence. An outstanding impact of the prophylactic treatment on the clinical course was observed in all the patients. Conversely, severe infections leading to the early death of the patient were observed when the prophylaxis was stopped, supporting the importance of early identification and treatment. Genetic analysis of the three unrelated patients showed the same three nucleotide deletion in the *MYD88* gene (Figure 1), which was already reported in patients from the same ethnicity, suggesting that this may result from a founder effect. Surprisingly, of all the eleven kindreds described so far, our three patients from unrelated families were all living in the same region in Italy. To our knowledge, only another patient has been reported in Italy, in the Lazio region [13]. Since this ethnic group is distributed in different Italian regions, with the highest representation in Lazio and Lombardia, we would have expected a higher incidence of cases in these regions. This evidence may support the hypothesis that the diagnosis of this rare condition may be underestimated due to the lack of identification of the warning signs that may be missed due to the disproportion between the severity of the condition and the symptoms and laboratory findings. Hence, this report highlights the importance of identifying tools for the screening of these severe but actionable diseases. NGS may represent an attractive modality for newborn screening in the field of IEIs, even though there are significant limitations that may hinder its clinical application, including the elevated costs and time for analysis and the uncertainty of genetic results. Therefore, since currently there are no available screening tools for this disease, a high level of alert should be kept in patients of Roma ethnicity with early onset recurrent or severe infections. Considering the frequency of this specific genetic alteration, direct Sanger sequencing may be used for a rapid screening in patients of Roma ethnicity presenting with a suggestive phenotype characterized by early onset invasive pyogenic infections with only mild signs of inflammation. Furthermore, although MyD88 deficiency is not listed in the International Union of Immunological Societies classification among the causes of Hyper-IgE syndromes, it should be considered in their differential diagnosis. Indeed, it should be kept in mind that, especially in very young children, where pathognomonic signs of Hyper-IgE syndromes may be absent, the clinical features of MyD88 deficiency and other Hyper-IgE syndromes may be overlapping since both the conditions may present with early onset invasive suppurative infections with a scarce inflammatory component. This is also supported by the observation that the NIH HIES score, calculated for our patients, was suggestive of a “possible” diagnosis. For this reason, targeted genetic panels requested for suspected Hyper-IgE syndromes should also include the *MYD88* gene. However, it should be noted that rarely IgE levels result as high as in disorders due to mutations in *STAT3* or *DOCK8* [14,15].

Although the pathogenesis of IgE increase in MyD88 deficient patients still remains to be elucidated, several mechanisms for this laboratory feature have been proposed in preclinical studies. First, the TLR4-MyD88 pathway leads to the production of IL-12 by dendritic cells and the promotion of Th1 differentiation. Thus, the impairment of IL-12 production has been hypothesized to entail a preferential Th2 differentiation in MyD88 deficient mice. However, this hypothesis is not supported by the evidence that Th1 response is not impaired in IL-12-deficient mice [16]. Another recent study observed that IgE from *Myd88*^−/−^ mice recognized a commensal bacterium, *Streptococcus azizii*, highly represented in the lungs of knock-out mouse models. In this model, the serum IgE levels were reduced by antibiotics against this microorganism (thus suggesting that some commensal bacteria may prime the Th2 response and IgE production [17].

Additionally, the three patients showed slightly decreased IgM levels, as also previously reported in a few patients in the literature [5]. Even though the mechanism leading to hypo-IgM has not been clarified, different studies suggest the role of MyD88 in B cell development and function [18,19,20]. Interestingly, activating MyD88 mutations have been associated with Waldenström Gammopathy, suggesting a specific role for MyD88 in IgM production [21].

Definitive treatment with HSCT has never been reported in MyD88 patients, whose clinical phenotype tends to ameliorate beyond teenage years, likely due to the contribution of adaptive immunity [22]. Moreover, as observed in our cohort, antibiotic prophylaxis and IgRT have a dramatic impact on survival, even though close monitoring of clinical events and treatment adherence in this ethnic group is often hampered by their lifestyle. Due to the severity of the clinical course (fatal meningitis in one of them and recurring serious deep-seated infections in the other two), we sought a related donor for our two young surviving patients, and we found identical HLA in the parents, supposedly favored by the high grade of inbreed among these subjects. This may increase the likelihood of identifying matched related donors among the parents and close relatives when deemed necessary.

## 5. Conclusions

This case series underscores the importance of keeping a high level of alert in patients of Roma ethnicity with early onset pyogenic and severe infections in whom a diagnosis of inborn error of immunity as MyD88 deficiency may be suspected. An early diagnosis and initiation of prophylaxis are pivotal due to the high mortality rate in early infancy. In particular, in this ethnic group, we observed an in-frame deletion, c.157_159del p.(Glu53del) in *MYD88*, already described in this nomad population, in which it may have a founder effect. Furthermore, being associated with increased IgE levels, this condition should be included in the differential diagnosis of Hyper-IgE syndromes.

## Figures and Tables

**Table 1:** Patients’ characteristics.

	Age of Onset	Age at Diagnosis	Genotype	Management	Age/Cause of Death	Ethnicity	Consanguinity
P1	7 months *Y. enterocolitica* terminal ileitis	2 years	Homozygous in-frame deletion c.157_159del p.(Glu53del)	Trimethoprim-sulfamethoxazole 6 mg/kg/od	7 years *Streptococcus pneumoniae* meningoencephalitis	Roma	Yes
P2	2 months *P. aeruginosa* endophthalmitis	8 years	Homozygous in-frame deletion c.157_159del p.(Glu53del)	Trimethoprim-sulfamethoxazole 6 mg/kg/od Amoxicillin 20 mg/kg/od IgRT 400 mg/kg/every 21 days	Patient alive	Roma	Yes
P3	4 months Sub-mandibular lymphadenopathy	23 months	Homozygous in-frame deletion c.157_159del p.(Glu53del)	Amoxicillin 20 mg/kg/od IgRT 400 mg/kg/every 21 days	Patient alive	Roma	Yes

Abbreviations. IgRT, immunoglobulin replacement therapy.

**Table 2:** Patients’ immunological features.

	Immunoglobulins	Specific Antibody Response	Total IgE	Immunophenotyping	NBT and DHR Tests	PHA and PKW Response	TLR Stimulation
P1	IgG 1390 mg/dL (462–1710), IgA 50.5 mg/dL (27–173), IgM 40.9 mg/dL (62–257)	HBsAb: present Anti-oneumococcal antibodies: absent	1190 IU/L (<200)	Normal	Normal	Normal	Low response
P2	IgG 1380 mg/dL (633–1016), IgA 92.8 mg/dL (41–315), IgM 55.6 mg/dL (56–261)	HBsAb: absent Anti-pneumococcal antibodies: absent	2014 IU/L (<200)	Normal	Normal	Normal	Low response
P3	IgG 1390 mg/dL (264–1509) IgA 92.8 mg/dL (17–178) IgM 55 mg/dL (48–337)	HBsAb: absent Anti-pneumococcal antibodies: absent	2000 IU/L (<200)	Normal	Normal	Normal	Low response

Abbreviations. HBsAb, Hepatitis B surface antibodies; NBT, Nitroblue tetrazolium chloride test; DHR, dihydrhodamine 123 test; PHA, phytohaemagglutinine mitogen; PKW, pokeweed mitogen; TLR, toll-like receptor.

## Data Availability

The data used in this study are available from the corresponding author, and the authors can share the information if there is a reasonable request.

## Abbreviations

DHR, dihydrhodamine 123 test; IEI, inborn errors of immunity; IgE, immunoglobulin E; IgRT, immunoglobulin replacement therapy; HLA, human leukocyte antigen; HSCT, hematopoietic stem cell transplant; NBT, Nitroblue tetrazolium chloride test; *MYD88*, Myeloid differentiation primary response gene 88; TLR, toll-like receptor; t-NGS, targeted next-generation sequencing.
